# NCBP1 enhanced proliferation of DLBCL cells via METTL3-mediated m6A modification of c-Myc

**DOI:** 10.1038/s41598-023-35777-2

**Published:** 2023-05-27

**Authors:** Sibo Meng, Yuan Xia, Mingying Li, Yuyan Wu, Dongmei Wang, Ying Zhou, Daoxin Ma, Jingjing Ye, Tao Sun, Chunyan Ji

**Affiliations:** 1 Department of Hematology, Qilu Hospital of Shandong University, Cheeloo College of Medicine, Shandong University, Jinan, 250012 Shandong People’s Republic of China; 2 Department of Medical Oncology, Qilu Hospital (Qingdao), Cheeloo College of Medicine, Shandong University, 758 Heifei Road, Qingdao, 266035 Shandong People’s Republic of China

**Keywords:** Cancer therapy, Haematological cancer

## Abstract

Diffuse large B-cell lymphoma (DLBCL) is malignant hyperplasia of B lymphocytes and standard care cannot satisfactorily meet clinical needs. Potential diagnostic and prognostic DLBCL biomarkers are needed. NCBP1 could bind to the 5ʹ-end cap of pre-mRNAs to participate in RNA processing, transcript nuclear export and translation. Aberrant NCBP1 expression is involved in the pathogenesis of cancers, but little is known about NCBP1 in DLBCL. We proved that NCBP1 is significantly elevated in DLBCL patients and is associated with their poor prognosis. Then, we found that NCBP1 is important for the proliferation of DLBCL cells. Moreover, we verified that NCBP1 enhances the proliferation of DLBCL cells in a METTL3-dependent manner and found that NCBP1 enhances the m^6^A catalytic function of METTL3 by maintaining METTL3 mRNA stabilization. Mechanistically, the expression of c-MYC is regulated by NCBP1-enhanced METTL3, and the NCBP1/METTL3/m^6^A/c-MYC axis is important for DLBCL progression. We identified a new pathway for DLBCL progression and suggest innovative ideas for molecular targeted therapy of DLBCL.

## Introduction

Diffuse large B-cell lymphoma (DLBCL) is characterized by malignant hyperplasia of B lymphocytes with an incidence of seven cases per 100,000 people per year^1^, accounting for almost 40% of all cases of non-Hodgkin’s lymphoma^2^. Less than 60% of DLBCL patients can be cured by rituximab-based chemotherapy (CHOP)^3^. Disappointingly, 30%-40% of patients relapse later or suffer from primary refractoriness and they often have a dismal outcome^4,5^. New therapeutic approaches may be the optimal management strategies for DLBCL, but thus far, the next-generation anti-CD20 antibodies ofatumumab, veltuzumab, and Obinutuzumab^6–8^ and novel therapies bortezomib^9^, lenalidomide^10^, ibrutinib^11^, or everolimus^12^ fail to provide additional benefit compared with R-CHOP. From this point of view, understanding the pathogenesis and mechanisms of DLBCL and searching for new therapeutic targets are urgently needed for the treatment of DLBCL patients.

The nuclear Cap-Binding Complex(CBC) associates with the nascent 5’ cap of RNA polymerase II transcripts to determine mRNA fate^13,14^. CBC consists of nuclear cap-binding protein 1 (NCBP1), 2 (NCBP2) and C17orf85 (NCBP3)^15^. It is common that NCBP1 binds to pre-mRNA in eukaryotic cells and patticipates in various RNA processing processes, such as pre-mRNA splicing, translation regulation, nonsense-mediated mRNA decay, mRNA export and RNP productive and destructive fate^16^. NCBP1 engaging with ribonucleoprotein particles (RNPs) can regulate cell growth and proliferation, adapt to environmental change^17,18^, escort antiviral mRNAs to combat the pathogens^19^, promote the translation of oncogenes^20^ and take part in chemotherapy resistance^21^ in vegetation, fungi, and human solid tumors. However, the fundamental abnormalities of NCBP1 underlying DLBCL remain elusive^22,23^.

N6-methyladenosine (m^6^A) is the most abundant RNA modification in eukaryotic cells^24,25^. m^6^A methylation is catalysed by a multicomponent methyltransferase complex. METTL3, a well-known RNA N^6^-methyladenosine writer, plays predominant roles in different biological processes^26,27^. For example, METTL3 regulates autophagy by enhancing m^6^A modification of ATG7 in osteoarthritis progression^28^; METTL3 mediates BCL2 stability via Ythdf1-mediated m^6^A modification and inhibits cell apoptosis^29^; METTL3 regulates lncRNA stability and improves CUL4B enter into the nucleus^30^. For DLBCL, only one report showed that METTL3 regulates PEDF m^6^A modification and is functionally implicated in DLBCL development^31^. In this study, we uncovered the oncogenic function of NCBP1 in DLBCL, proved that NCBP1 increases METTL3 mRNA transcript stabilization, and verified that NBCP1 impacts METTL3-mediated m^6^A modification of c-MYC in the forthcoming tumorigenesis of DLBCL. Our study addresses unmet clinical needs by gaining some insights into the function of NCBP1 in DLBCL.

## Methods

### Cell lines and cultures

SU-DHL-4 cells and DB cells (American Type Culture Collection, ATCC) were cultured in RPMI-1640 medium containing 10% fetal bovine serum, 100 U/mL penicillin and 100 µg/mL streptomycin, and maintained in a humidified 5% (v/v) CO_2_ atmosphere at a temperature of 37 °C. Cells stably expressing NCBP1 or METTL3 and control cells were subjected to viral transfection and puromycin pressure.

### Quantitative real-time PCR (RT‒qPCR)

Cells were lysed and extracted into total RNA using TRIzol (Invitrogen). RNA was reverse transcribed into cDNA using an RTase cDNA Synthesis Kit (Takara, Japan). After that, Applied Biosystem 7900HT System (ABI) was used to quantitatively analyze the mRNA expression of genes by RT-qPCR. Each sample was amplified in a 10 μL reaction volume with specific primers (Supplementary Table S1), SYBR Green PCR Master Mix (Toyobo, Japan) and RNase free water. In this study, GAPDH was applied as an internal control.

### Western blotting

Protein solubilization buffer was used for cell lysis. Protein extracts were performed according to the manufacturer’s instructions of Total Protein Extraction Kit (BestBio, Shanghai, China). SDS‒PAGE was using for separating the proteins, after that, proteins were transferred to nitrocellulose membranes (Millipore). Antibodies used in our study were mainly anti-GAPDH (Abways, AB0037), anti-NCBP1(Abcam ab154532, Proteintech 10349-1-AP), anti-METTL3 (Abcam ab195352, BETHYL A301-568A) and anti-MYC (Proteintech 10828-1-AP, Cell Signaling Technology #9402). GAPDH was served as a loading control. Finally, membranes were probed by HRP-conjugated secondary antibody (Servicebio GB23302) and visualized by using chemiluminescent reagents (Millipore, Merck, Darmstadt, Germany).

### Patient samples

Lymphoma tissue samples from 50 DLBCL patients were collected from needle biopsy or lymph node resection at Qilu Hospital of Shandong University, China. 14 control samples were collected from inflammatory lymph nodes. All primary samples were collected and used with informed consent and under the supervision of the Medical Ethics Committee of Qilu Hospital of Shandong University. Immunohistochemical detection of METTL3 and c-MYC was performed. RNA was extracted from paraffin-embedded sections, and RT‒qPCR was performed to evaluate the relationship between survival and NCBP1/METTL3/c-MYC expression.

### Lentiviral transduction

Lentiviral constructs repressing NCBP1 and METTL3 were purchased from Genechem (Shanghai, China). siRNA targeting NCBP1, METTL3, c-MYC and the corresponding controls were purchased from GenePharma (Supplementary Table S2). After efficiency verification, the interference fragment was sent to the company for lentivirus packaging. For transient transfection, cells were transfected using Lipofectamine 2000 (Invitrogen) following the manufacturer's recommendations for 24 h. For stable transfection, cells were transfected using lentivirus and selected using 1 μg/ml puromycin.

### Proliferation CCK-8 assay

A total of 5000 cells were seeded in 96-well plates and cultured in 100 μl of complete medium for 4 days. To assess proliferation, 10 μL CCK-8 (BestBio, Shanghai, China) was added into each well at indicated time point. Next, plates were incubated in 37 °C for 2–4 h and the absorbance signal at 450 nm was measured by using a microplate reader (Thermo Scientific).

### Flow cytometry of EdU

Lentivirus-transduced cell suspensions were washed twice using PBS containing 1% FBS. Then, according to standard protocols, the cells were incubated with EdU Component A for 4 h. After that, cells were fixed and permeabilized with Component C and D, and stained using Component E, B, and F. Finally, the stained cells were sieved and prepared for EdU signal detection with BD FACSAria III flow cytometer (BD Biosciences, San Jose, CA, USA). Further analysis of flow data were conducted by using Kaluza software.

### RNA sequencing (RNA-seq) and data analysis

RNA extraction upon NC and shNCBP1 SU-DHL-4 cells was performed according to the sequencing sample requirements. Briefly, raw reads were quality filtered and then trimmed. Cleaned reads were then mapped to GRCh38. Further analysis was performed using the proper pairs with both reads mapping to the genome. R package DESeq2 (v 1.20.0) were applied to analyze the differentially expressed transcripts with a false discovery rate (FDR) cut-off of 0.05. Meanwhile, the exon counts for differentially expressed transcripts were compared and measured using custom scripts in R.

### Immunohistochemistry

For immunohistochemical assessment, lymphoid tissue sections from DLBCL patients and controls were subjected to heat-induced epitope retrieval according to standard protocols of a histochemical kit (ZSGB-BIO). Then, primary antibodies anti-NCBP1(Abcam ab154532, Proteintech 10349-1-AP), anti-METTL3(Abcam ab195352, BETHYL A301-568A), and anti-c-MYC (Proteintech 10828-1-AP, Cell Signaling Technology #9402) were applied for a 4 °C overnight incubation. Then, incubation with HRP-conjugated secondary antibodies were performed, following with DAB staining. Finally, the slides were visualized and photographed using a light microscope (Nikon, Ni–U).

### Clonogenicity assay

In this method, lentiviral transduction SU-DHL-4 cells were incubated in a sterile six-well plate for 7 days. When the incubation period was over, the number of colonies was calculated using a microscope.

### Methylated RNA immunoprecipitation (MeRIP)-qPCR

Total RNA was extracted from lentiviral transduction SU-DHL-4 (NCBP1 and NC) cells using TRIzol (Invitrogen). Based on the manufacturer’s instructions, anti-m^6^A antibody (ab151230) and IgG previously coated on protein A/G beads were incubated with 100 μg of RNA from each group overnight at 4 °C. Subsequently, m^6^A bound RNA was eluted from the beads and extracted to detect the levels of methylated c-MYC RNA by using RT-qPCR.

### m^6^A dot blot

Total RNA was isolated from SU-DHL-4 cells using Trizol (Invitrogen) according to the manufacturer’s instructions. The quality of RNA was analyzed using RNA electrophoresis. 200 ng of RNA from all samples was UV cross-linked onto a wool membrane, blocked and incubated with m^6^A antibody (ab195352, 1:1000). After secondary incubation, the blots were visualized to acquire the m^6^A levels of all samples by using chemiluminescent reagents (Millipore, Merck, Darmstadt, Germany).

### RNA stability (actinomycin D assays)

Lentivirus transfected SU-DHL-4 cells were seeded at 1 × 10^5^ cells per well in six-well plates. 24 h later, 5 μg/ml actinomycin D (Act D, Sigma) were added into SU-DHL-4 cells, and cells were harvested at the indicated times (2 h, 4 h, 6 h). For evaluation of RNA stability, RT-qPCR was performed to access the remaining RNA levels as normalized to the control group at 0 h.

### Statistical analyses

Mean ± standard deviation (SD) were measured and presented using Graphpad Prism 8.0. Unpaired Student’s t-test were applied to access the differences between 2 groups. For 3 or more groups, the differences were measured using one-way or two-way ANOVA. In case of unequal variances, Mann‒Whitney U test was applied. Survival in DLBCL patients was represented with Kaplan‒Meier curves using R software. Statistically significance was accepted as P < 0.05.

### Ethics approval and consent to participate

No animal studies were involved in our research. The clinical specimens were conducted with permission from the Ethics Committee of Qilu Hospital, Jinan, China. The study was carried out in accordance with the Declaration of Helsinki and all patients signed informed consent forms before sampling.

## Results

### NCBP1 was overexpressed in the lymph nodes of DLBCL patients and related to their poor prognosis

**Figure 1:**
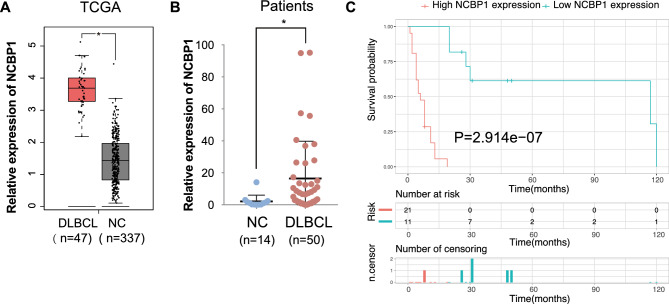
NCBP1 was overexpressed in the lymph nodes of DLBCL patients and was related to their poor prognosis. (**A**) NCBP1 expression in DLBCL tumors and normal tissues from the GEPIA database. (**B**) NCBP1 expression was measured in 50 DLBCL tissues and 14 inflammatory lymph nodes by quantitative RT‒PCR. (**C**) Overall survival of patients grouped by NCBP1 expression as either high (red) or low (blue) in DLBCL patients. High NCBP1 expression was considered to correlate with a short survival time. The results represent the mean ± SD; *P < 0.05; **P < 0.01, ***P < 0.001.

The expression of NCBP1 correlated with poor survival and short DFS in DLBCL in the TCGA database. To evaluate the role of NCBP1 in DLBCL patients, we studied the expression of NCBP1 in DLBCL from the GEPIA database and found that NCBP1 was more highly expressed in DLBCL tissues than in normal tissues (P < 0.05) (Fig. 1A). Then, we compared NCBP1 expression in 50 DLBCL tissues and 14 inflammatory lymph nodes diagnosed in Qilu Hospital by using RT‒qPCR. The results showed that NCBP1 was expressed at significantly higher in tumor tissue than in inflammatory lymph nodes (P = 0.0029) (Fig. 1B). To further verify the role of NCBP1 in DLBCL, we used Kaplan‒Meier analysis to compare the overall survival of 50 DLBCL patients with high and low/medium expression of NCBP1 (cut off = 50%) collected at Qilu Hospital. Due to the fact that some of the patients were lost to follow-up during survival statistics, only 32 patients were counted for their survival data (Fig. 1C). DLBCL patients with high expression of NCBP1 showed a significantly shorter survival time than patients with low/medium expression (P < 0.001).

### NCBP1 promoted proliferation of DLBCL cells in vitro

**Figure 2:**
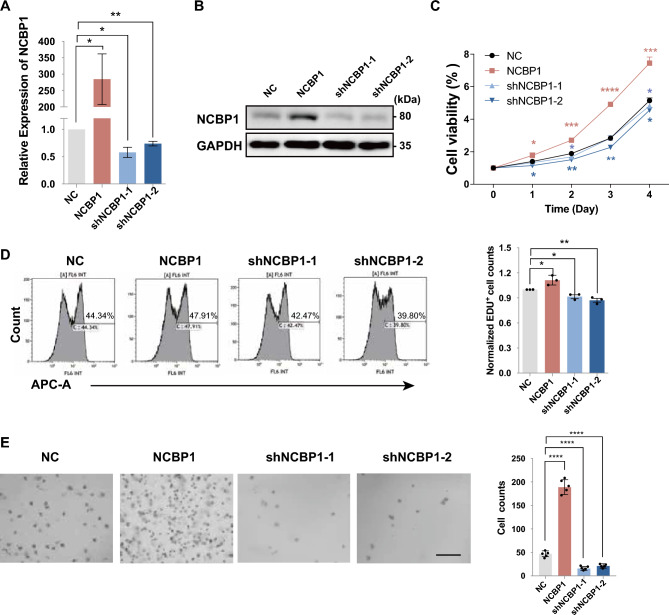
NCBP1 promoted proliferation of DLBCL cells in vitro. (**A**,**B**) Efficiency of NCBP1 in lentiviral transduction SU-DHL-4 cells was confirmed by RT‒qPCR and western blotting analysis on the RNA and proteins. (**C**) Cell viability of SU-DHL-4 cells following transfection was determined by CCK8 assay at the indicated time-points. (**D**) The cell proliferation of SU-DHL-4 cells following transfection was evaluated by EdU. (**E**) Semisolid culture was conducted to analyze the effects of NCBP1 on the proliferation potential of SU-DHL-4 cells. The data are presented as the mean ± SD. Statistically significant differences were defined as follows: *P < 0.05; **P < 0.01, ***P < 0.001.

To investigate whether NCBP1 affects the proliferation of DLBCL cells, SU-DHL-4 cells and DB cells with upregulated or downregulated NCBP1 expression were cultured to the logarithmic growth stage. mRNA and proteins were isolated to accomplish RT‒qPCR and western blot analysis for NCBP1 regulating efficiency (Fig. 2A,B and Supplementary Fig. S1A,B). Considering that shRNA has off-target effects, we used two different fragments expression suppressor strain to avoid adverse effects caused by their off-target. However, protein overexpression did not have the similar effect, so we only set up one group in the overexpression strain. Then, we performed a CCK-8 assay to determine the effects of NCBP1 on SU-DHL-4 cell and DB cell viability (Fig. 2C and Supplementary Fig. S1C). The results showed that downregulating NCBP1 significantly inhibited cell viability at 24, 48, 72 and 96 h (P < 0.05) compared with the effects of transfection with NC. Additionally, a significant increase in cell viability was also observed after transfection with NCBP1 overexpression lentivirus (P < 0.05). Furthermore, EdU proliferation analysis by flow cytometry was conducted, and as expected, NCBP1 overexpression/silencing in DLBCL cells increased/decreased the proliferation of DLBCL cells (Fig. 2D and Supplementary Fig. S1D). Additionally, in the semisolid culture assay, silencing NCBP1 expression resulted in inhibition of the formation of the SU-DHL4 cell community, and a greater cell community was observed in the NCBP1 overexpression group (Fig. 2E). In summary, NCBP1 significantly promoted the proliferation of DLBCL cells in vitro.

### NCBP1 increased the expression of METTL3 in DLBCL cells

**Figure 3:**
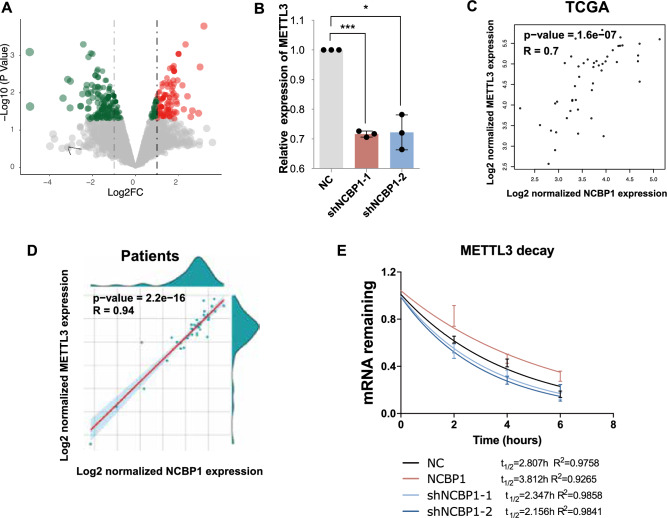
NCBP1 increased the expression of METTL3 in DLBCL cells. (**A**) Volcano plot showing that the differentially expressed gene METTL3 was related to shNCBP1 in SU-DHL-4 cells. (**B**) RT‒qPCR verified that METTL3 was downregulated by shNCBP1. (**C**) METTL3 expression was significantly related to NCBP1 (r = 0.7; P < 0.0001) in DLBCL tissue (data from the GEPIA database). (**D**) The relationship between NBCP1 and METTL3 mRNA levels by RT‒qPCR analysis in DLBCL tumor samples. (**E**) The half-life (T1/2) of METTL3 mRNA in SU-DHL4 cells transfected with Lv-oeNCBP1, Lv-shNCBP1 and Lv -NC (the control lentivirus). Statistically significant differences were defined as follows: *P < 0.05; **P < 0.01, ***P < 0.001.

To investigate how NCBP1 affected the proliferation of DLBCL, we performed RNA-seq by using SU-DHL-4 cells transfected with NCBP1 silencing vectors. The results showed that 223 genes were upregulated in shNCBP1 SU-DHL-4 cells, and 163 genes were downregulated. Among these downregulated genes, METTL3 was one of the predicted downstream genes and had significant expression differences in the differential gene volcano plot (Fig. 3A). Then, we proved that METTL3 mRNA expression was significantly decreased in SU-DHL-4 cells transfected with shNCBP1 compared with that in the NC group by RT‒qPCR (Fig. 3B). These results suggest that METTL3 might act as a downstream target of NCBP1 in DLBCL. Furthermore, we found that METTL3 was significantly positively correlated, albeit moderately, with NCBP1 using linear regression analysis in DLBCL tumor tissues from the GEPIA data set (r = 0.7; P < 0.0001) (Fig. 3C). Similarly, from our clinical DLBCL tissue samples, we found that the mRNA expression of upregulated NCBP1 was positively correlated with high METTL3 expression levels (Fig. 3D). Next, as NCBP1 specifically bound to the Cap structure and mediated gene expression, we carried out an RNA stability assay by incubating cells with actinomycin-D to block transcription and then measured the time required until RNA levels were reduced to 50% of the original abundance. The results showed that NCBP1 combined with the cap of METTL3 mRNA and increased the stability of its RNA (Fig. 3E). In summary, NCBP1 enhanced the expression of METTL3 in DLBCL cells.

### NCBP1 enhanced the proliferation of DLBCL cells via METTL3

**Figure 4:**
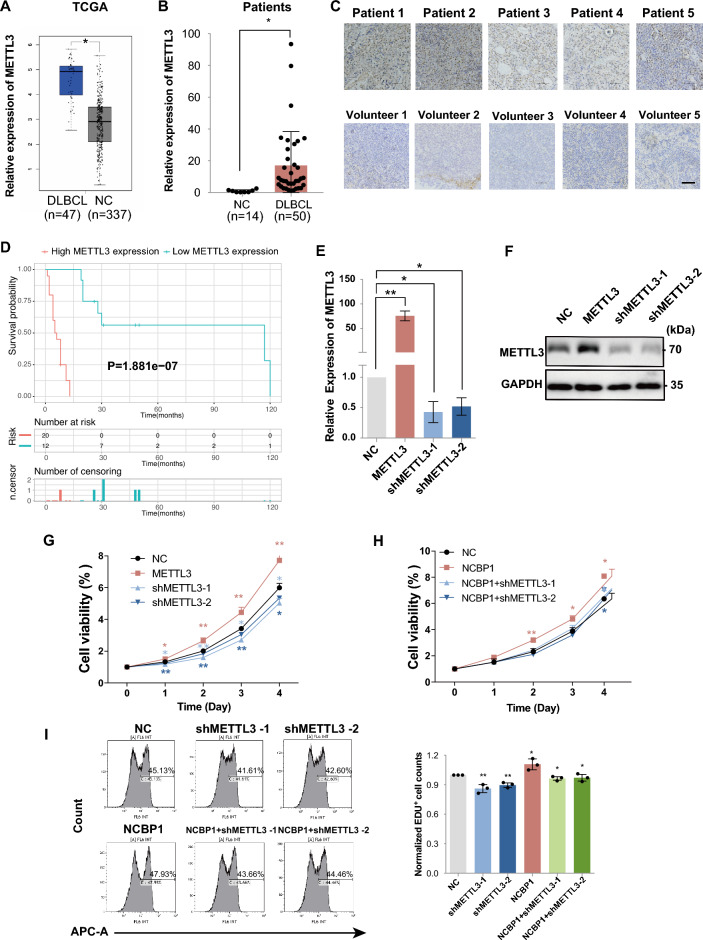
NCBP1 enhanced the proliferation of DLBCL cells via METTL3. (**A**) METTL3 expression in DLBCL tumors and normal tissues from the GEPIA database. (**B**) METTL3 expression in 50 DLBCL tissues and 14 inflammatory lymph nodes measured by RT‒qPCR. (**C**) Representative images show the differential expression of METTL3 in DLBCL tissue and inflammatory lymph nodes by immunohistochemistry (IHC). (**D**) Overall survival of patients grouped by METTL3 expression in DLBCL patients, high (red) or low (blue) METTL3. (**E**,**F**) The efficiency of METTL3 in lentiviral transduction SU-DHL-4 cells was confirmed by RT‒qPCR and western blotting of RNA and proteins. (**G**,**H**) Cell viability of SU-DHL-4 cells following transfection was determined by CCK8 assay at the indicated time points. (**I**) The proliferation of SU-DHL-4 cells following transfection was evaluated by EdU. The data are presented as the mean ± SD. Statistically significant differences were defined as follows: *P < 0.05; **P < 0.01, ***P < 0.001.

Before studying whether NCBP1 promotes DLBCL via METTL3, we systematically studied the impact of METTL3 on DLBCL. The METTL3 mRNA expression data from the TCGA database (Fig. 4A) and our clinical samples (Fig. 4B) showed that METTL3 was highly expressed in DLBCL. Similarly, immunohistochemical staining (Fig. 4C and Supplementary Fig. S2A) proved that tumor tissues had higher METTL3 protein expression. Furthermore, we used Kaplan‒Meier analysis to compare the overall survival of 50 DLBCL patients with high or low/medium expression of METTL3 (cut off = 50%) collected in Qilu Hospital (Fig. 4D). Some of the patients were lost to follow-up during survival statistics, resulting in a decrease in the total number of patients. DLBCL patients with high METTL3 expression showed significantly lower survival than patients with low/medium METTL3 expression (P < 0.001). Then, METTL3 overexpression or knockdown lentiviruses were constructed, and METTL3 regulation efficiency was validated by using RT‒qPCR and western blotting (Fig. 4E,F and Supplementary Fig. S1E,F). The results of CCK-8 assays showed that increasing METTL3 expression significantly enhanced proliferation and that silencing METTL3 expression led to inhibition of DLBCL cell proliferation comparing with the METTL3 over expression group (Fig. 4G and Supplementary Fig. S1G). All the results proved that METTL3 was pivotal for DLBCL. Next, we found that shMETTL3 partially blocked the increasing effects of NCBP1 overexpression on DLBCL cell proliferation (Fig. 4H and Supplementary Fig. S1H). Similarly, the flow cytometry EdU assay also showed that METTL3 knockdown blocked the enhancement of DLBCL cell proliferation by NCBP1 overexpression (Fig. 4I). In summary, these results confirmed that NCBP1 acted as a METTL3 dependent oncogene in DLBCL pathogenesis.

### METTL3 enhanced the proliferation of DLBCL cells by increasing c-MYC expression

**Figure 5:**
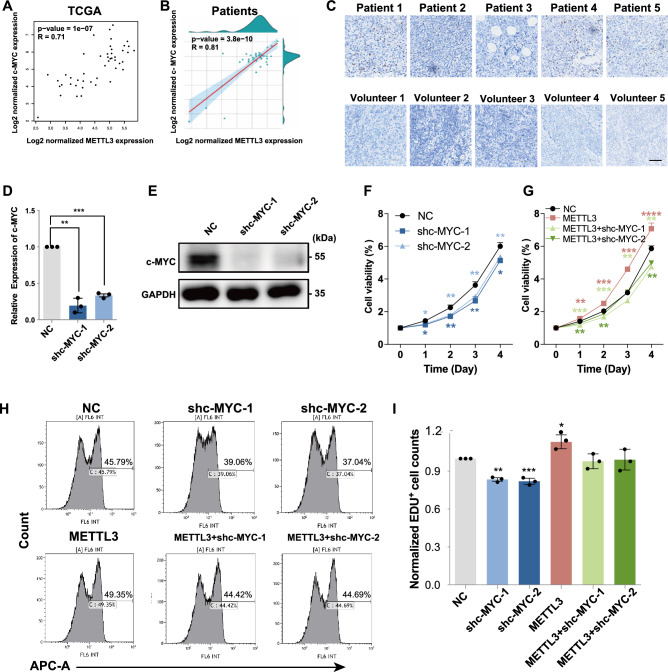
METTL3 enhanced the proliferation of DLBCL cells by increasing c-MYC expression. (**A**) Positive correlation between the expression of c-MYC and METTL3. (**B**) DLBCL specimen showed c-MYC mRNA was related to METTL3. (**C**) Immunohistochemistry staining images of c-MYC expression in DLBCL tissue and inflammatory lymph nodes. (**D**,**E**) The efficiency of c-MYC in lentiviral transduction SU-DHL-4 cells was confirmed by RT‒qPCR and western blotting of RNA and proteins. (**F**,**G**) Cell viability of SU-DHL-4 cells following transfection was determined by CCK8 assay at the indicated time-points. (**H**,**I**) The cell proliferation of SU-DHL-4 cells following transfection was evaluated by EdU. Statistically significant differences are defined as follows: *P < 0.05; **P < 0.01, ***P < 0.001.

c-MYC is considered a modification target of METTL3 and plays an important role in many tumors. To investigate whether METTL3 mediates the proliferation of DLBCL cells via c-MYC, we first studied mRNA expression data from the GEPIA dataset and our clinical samples. The results showed that the expression of c-MYC was significantly positively correlated with METTL3 using linear regression analysis in DLBCL (Fig. 5A,B). Additionally, immunohistochemical staining showed that DLBCL tissues had higher c-MYC protein expression levels (Fig. 5C and Supplementary Fig. S2B). Then, we downregulated c-MYC expression in SU-DHL4 cells and DB cells (Fig. 5D,E and Supplementary Fig. S2C,D) and found that METTL3-enhanced proliferation of DLBCL cells could be blocked by silencing c-MYC by CCK-8 (Fig. 5F,G and Supplementary Fig. S2E,F) and EdU incorporation assays (Fig. 5H,I). Taken together, our results demonstrated that c-MYC was a functionally important target of METTL3 and was important for NCBP1-mediated promotion of DLBCL cell proliferation.

### NCBP1 increased the m^6^A modification of c-MYC mRNA

**Figure 6:**
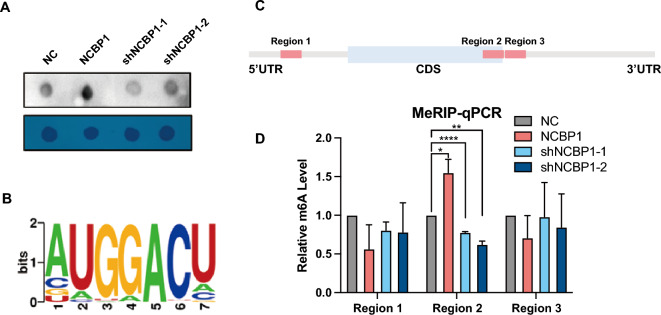
NCBP1 increased the m^6^A modification of c-MYC mRNA. (**A**) NCBP1 affected the degree of m^6^A methylation, and methylene blue (MB) staining was used as a loading control. (**B**) c-MYC modification site in previous reports. (RNA Modification Base, http://rna.sysu.edu.cn/rmbase/). (**C**) c-MYC mRNA transcript high abundance of potential m^6^A modification. (**D**) The m^6^A abundances on c-MYC mRNA transcripts in SU-DHL-4 cells as detected by MeRIP-qPCR, which showed m^6^A modification in specific regions of c-MYC. Statistically significant differences are defined as follows: *P < 0.05; **P < 0.01, ***P < 0.001.

METTL3 was reported to mediate RNA methylation. To investigate whether NCBP1 enhanced c-MYC RNA m^6^A methylation by increasing the expression of METTL3, we performed m^6^A dot blotting, and the results showed that the expression of NCBP1 was related to the m^6^A methylation levels in SU-DHL-4 cells (Fig. 6A). Then, we analyzed the modification of m^6^A to obtain the DRACH motif (Fig. 6B) using the m^6^A site recorded in the RMBase v2.0 database (RNA Modification Base, http://rna.sysu.edu.cn/rmbase/). Furthermore, we summarized the RNA fragments around the m^6^A site and selected three regions with a high abundance of potential m^6^A modifications from the 5'UTR, the CDS near the 3'UTR and the 3'UTR of the c-MYC mRNA transcript (Fig. 6C). Finally, three pairs of primers for the three regions were designed, and MeRIP-qPCR was carried out. The results showed that the amount of the CDS near the 3’ UTR of c-MYC mRNA that was pulled down by the m^6^A antibody was significantly increased/decreased when NCBP1 was overexpressed/knocked down in SU-DHL-4 cells (Fig. 6D), indicating that NCBP1 methylated the CDS near the 3ʹ UTR of c-MYC mRNA by METTL3. In the same time, the expression of NCBP1 and c-Myc was up-regulated in the NCBP1-overexpressed cells and down-regulated in the NCBP1-suppressed cells (Supplementary Fig. S2G–J). These results give us further confirmation that NCBP1 enhanced c-MYC RNA m^6^A methylation by increasing the expression of METTL3.

## Discussion

Previous studies have revealed that NCBP1 is concentrated in nonspecific RNA processing events, transcript nuclear export and translation. For example, NCBP1 assembles on pre-mRNAs with high affinity and increases the efficiency of small nuclear RNP(snRNP) core spliceosome components U1 to remove introns^32^. NCBP1 interacts with the EJC complex, exporting mature mRNA from the nucleus to the cytoplasm^33^. NCBP1 participates in the 3’UTR processing reaction by increasing poly(A) site cleavage complex stability^34^. NCBP1 binds with UPF1 to form the SURF complex and recruits SMG7, leading to nonsense-mediated mRNA decay^35^. Based on the NCBP1 pivotal role of NCBP1 in pre-mRNA biogenesis, aberrant NCBP1 expression is involved in tumour pathogenesis. For example, high expression of NCBP1 contributes to progression, wound healing ability, migration and epithelial-mesenchymal transition in NSCLC via the NCBP1-CUL4B oncoprotein axis^36^. NCBP1 cooperates with RHA to form a noncanonical cap-binding complex in activating mTOR-independent translation of tumour suppressor JUND mRNA in dysregulation of the immune response^37^. However, the influence of NCBP1 in DLBCL has not yet been reported. In this study, we showed that NCBP1 enhanced the proliferation of DLBCL cells by increasing the expression of METTL3, which deepened our understanding of NCBP1.

Researchers have discovered that the expression of METTL3, a well-known RNA N6-methyladenosine catalytic core, is upregulated in most cancers, such as bladder cancer^38^, ovarian carcinoma^39^, breast cancer^40^, gastric cancer^41^, and NSCLC^42^. Moreover, METTL3 modulates a significant number of cellular processes, including the cell cycle, cell proliferation, cell apoptosis, cell migration and invasion, and cell differentiation^43–46^. Many targeting molecules have been reported for MELTT3; however, studies on the regulatory mechanisms of METTL3 expression are just beginning. For example, HBXIP inhibits miRNA let-7g expression and relieves the inhibition of METTL3 expression in breast cancer^40^. miR-600^47^ and miR-33a^42^ directly inhibit METTL3 targeting EGFR, subsequently, lowering the levels of the phosphorylated form of AKT and NSCLC cell proliferation. miR-4429 increases the expression of SEC62 by targeting METTL3 to promote GC cell proliferation^48^. Our study suggests that NCBP1 expression positively correlates with METTL3 and stabilizes it by decreasing RNA decay. This is an innovative idea for the molecular targeted therapy of DLBCL and a new understanding of NCBP1 in the tumor regulatory mechanism.

In recent years, the relationship between m^6^A and lymphoma has been revealed preliminarily. For example, piRNA-30473 enhances m^6^A methylation of hexokinase 2 via the m^6^A methylase WTAP to promote the progression of DLBCL^49^. Another m^6^A writer, METTL3, promotes DLBCL progression by regulating the m^6^A level of PEDF^31^. The only article that confirmed that m^6^A modification has a significant regulatory effect on DLBCL. Different m^6^A writers, including METTL3 and WTAP, can regulate DLBCL through different target molecules. It is clear that our understanding of the relationship between m^6^A and DLBCL is still preliminary. Our study proves that METTL3 promotes the proliferation of DLBCL cells by regulating the m^6^A methylation of c-MYC, which further clarified the mechanisms of m^6^A methylation system abnormalities in DLBCL and revealed that substrates for METTL3 in DLBCL have obvious diversity.

## Conclusions

In conclusion, our study elucidates the regulatory role of NCBP1 in tumorigenesis and development, and it is perhaps a potential novel diagnostic and prognostic biomarker in DLBCL and can be used as a therapeutic target. In addition, we uncovered the regulatory mechanism of METTL3 in DLBCL, enriching our understanding of the m^6^A modification system in DLBCL.

## Supplementary Information

Supplementary Information 1.

Supplementary Information 2.

Supplementary Information 3.

Supplementary Information 4.

Supplementary Information 5.

Supplementary Information 6.

Supplementary Information 7.

Supplementary Information 8.

## Data Availability

Materials described in the manuscript, including all relevant raw data, will be freely available to any researcher wishing to use them for non-commercial purposes, without breaching participant confidentiality. All relevant data in this study could bu requested from the corresponding author CYJ (jichunyan@sdu.edu.cn).
