# Supplementary material for: NCBP1 enhanced proliferation of DLBCL cells via METTL3-mediated m6A modification of c-Myc
Source: Sci Rep. 2023 May 27;13:8606. doi: 10.1038/s41598-023-35777-2 (PMC10224985; doi:10.1038/s41598-023-35777-2)
Supplement: Supplementary file 1 — Supplementary Information 1. [file 41598_2023_35777_MOESM1_ESM.pdf]

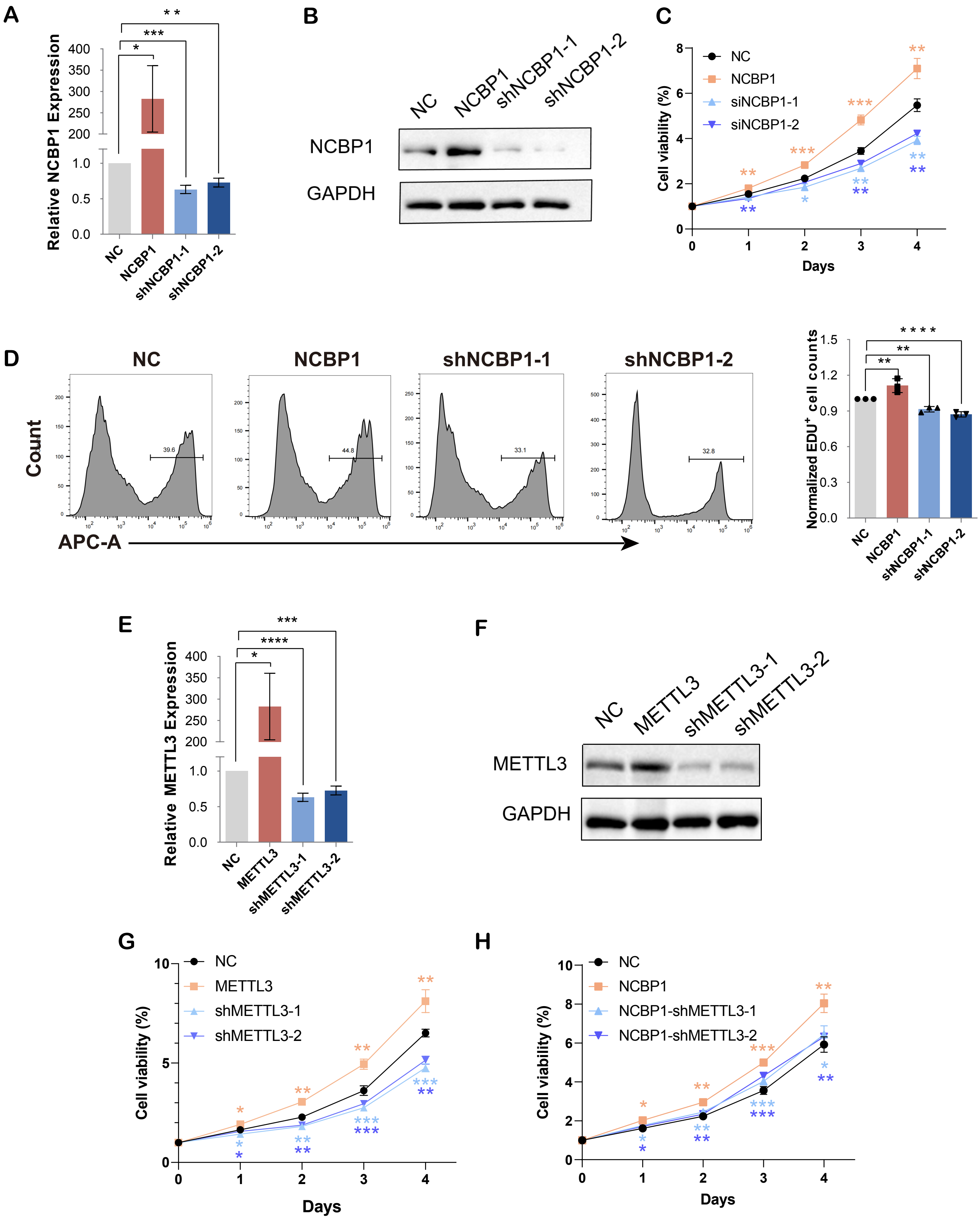

SUPPLEMENTARY FIGURE 1. NCBP1 promoted proliferation of DB cell line in vitro.
A, B, Efficiency of NCBP1 in lentiviral transduction DB cells was confirmed by RT-qPCR and western blotting analysis on the RNA and proteins. C, Cell viability of DB cells following transfection was determined by CCK8 assay at the indicated time-points. D, The cell proliferation of DB cells following transfection was evaluated by EdU. E, F, The efficiency of METTL3 in lentiviral transduction DB cells was confirmed by RT–qPCR and western blotting of RNA and proteins. G, H, Cell viability of DB cells following transfection was determined by CCK8 assay at the indicated time points.
