# Supplementary material for: NCBP1 enhanced proliferation of DLBCL cells via METTL3-mediated m6A modification of c-Myc
Source: Sci Rep. 2023 May 27;13:8606. doi: 10.1038/s41598-023-35777-2 (PMC10224985; doi:10.1038/s41598-023-35777-2)
Supplement: Supplementary file 2 — Supplementary Information 2. [file 41598_2023_35777_MOESM2_ESM.pdf]

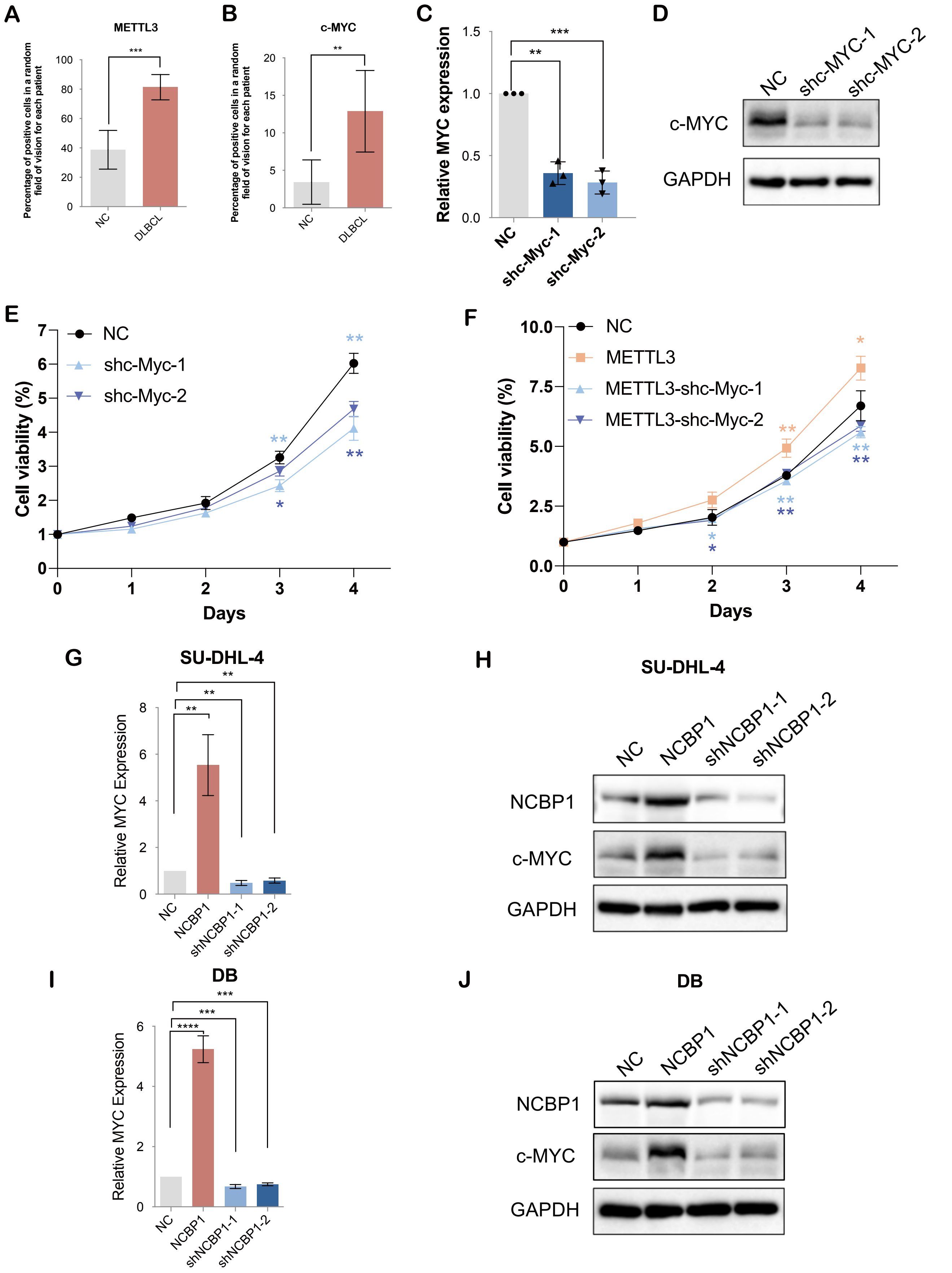

SUPPLEMENTARY FIGURE 2. NCBP1 promoted the proliferation of DLBCL cells by increasing c-MYC expression.
A, B, The percentage of positive cells of METTL3 and c-MYC in DLBCL tissue and inflammatory lymph nodes. C, D, The efficiency of c-MYC in lentiviral transduction DB cells was confirmed by RT–qPCR and western blotting of RNA and proteins. E, F, Cell viability of DB cells following transfection was determined by CCK8 assay at the indicated time-points. G, H,The relationship between NBCP1 and c-MYC mRNA levels by RT–qPCR analysis in in SU-DHL4 cells and DB cells.
