# Supplementary material for: NCBP1 enhanced proliferation of DLBCL cells via METTL3-mediated m6A modification of c-Myc
Source: Sci Rep. 2023 May 27;13:8606. doi: 10.1038/s41598-023-35777-2 (PMC10224985; doi:10.1038/s41598-023-35777-2)
Supplement: Supplementary file 3 — Supplementary Information 3. [file 41598_2023_35777_MOESM3_ESM.pdf]

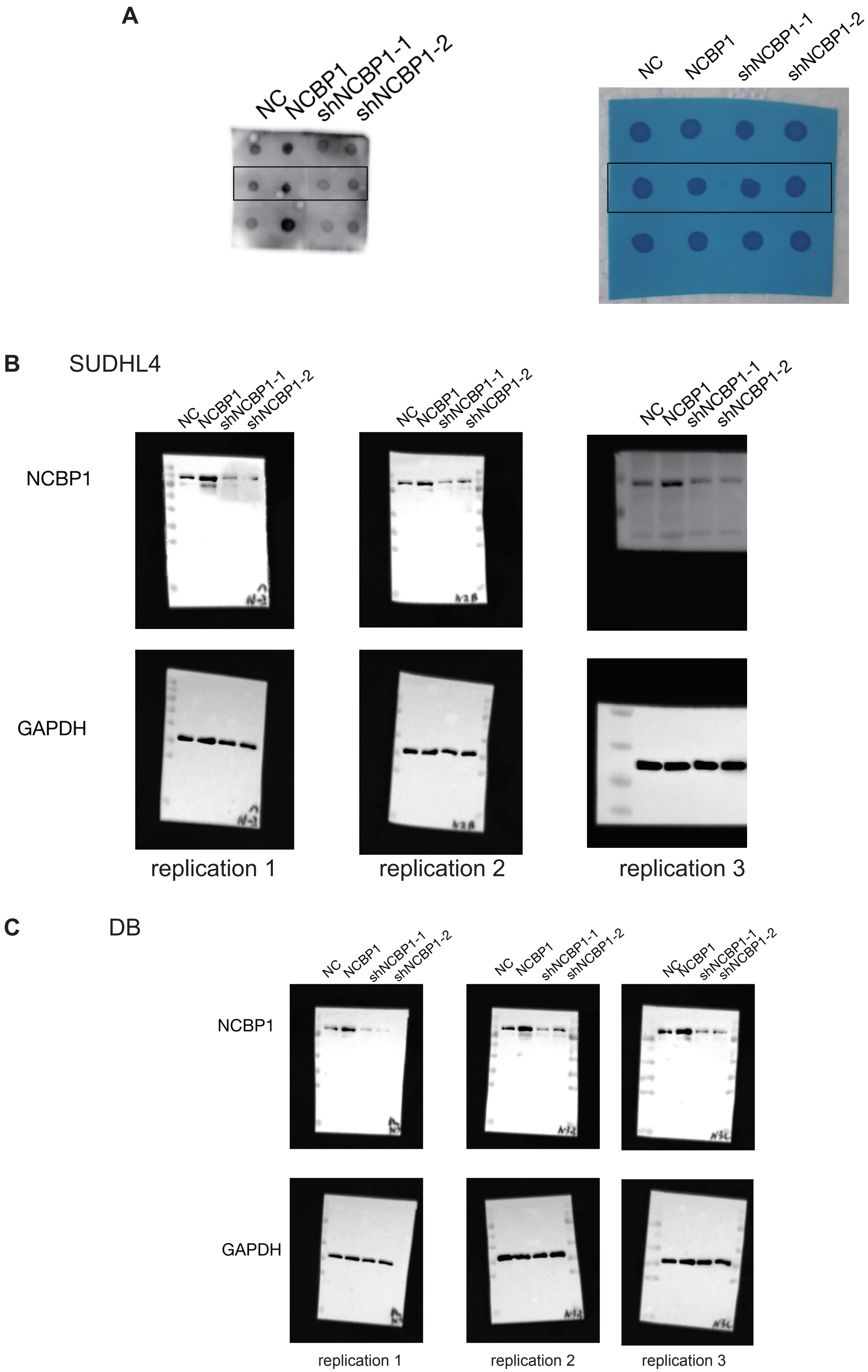

Supplementary Figure 3. Full-length gels and blots.
A, Raw blots of figure 6A. B-C, Full-length gels of figure 2B and S1B.
