# Supplementary figures and images for: NCBP1 enhanced proliferation of DLBCL cells via METTL3-mediated m6A modification of c-Myc
Source: Sci Rep. 2023 May 27;13:8606. doi: 10.1038/s41598-023-35777-2 (PMC10224985; doi:10.1038/s41598-023-35777-2)

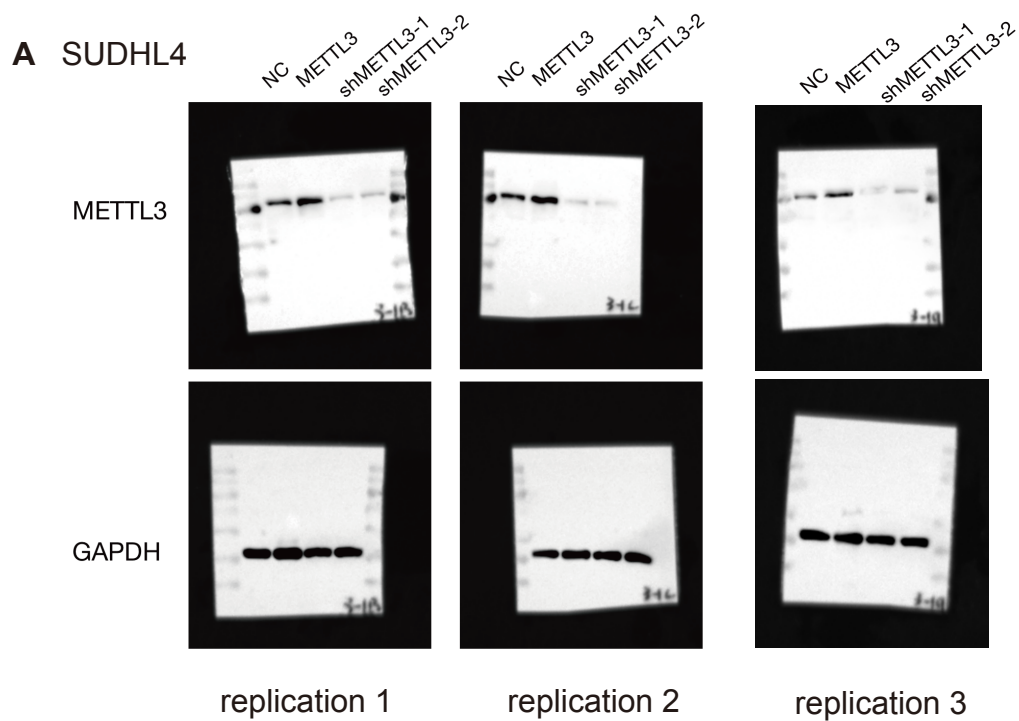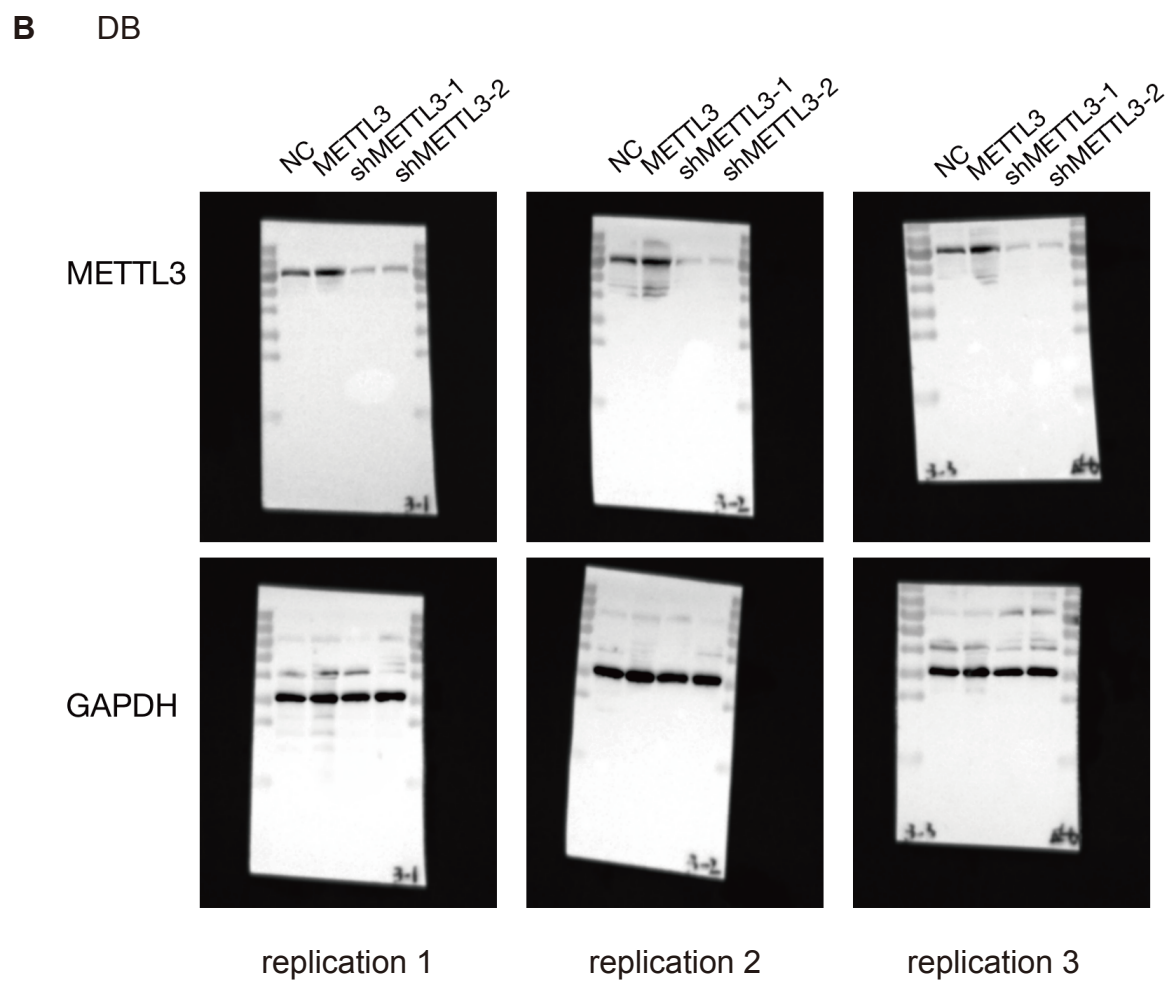

**Supplementary Figure 4. Full-length gels and blots.**  
A-B, Full-length gels of figure 4F and S1F.

Supplement: Supplementary file 4 — Supplementary Information 4. [file 41598_2023_35777_MOESM4_ESM.pdf]

## A SUDHL4

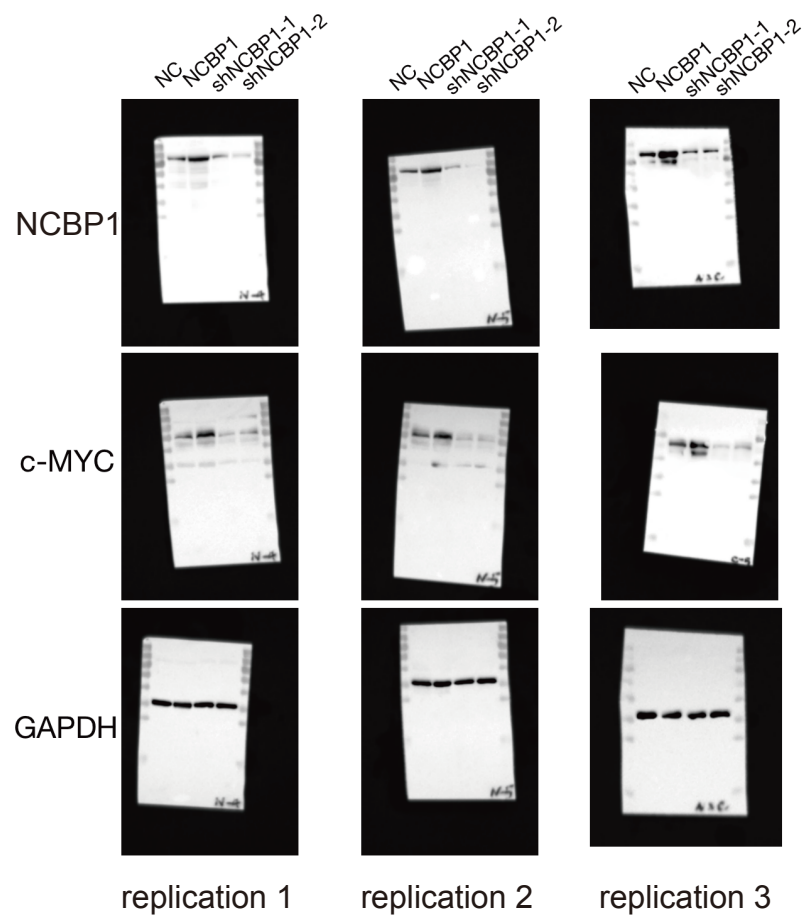

## B DB

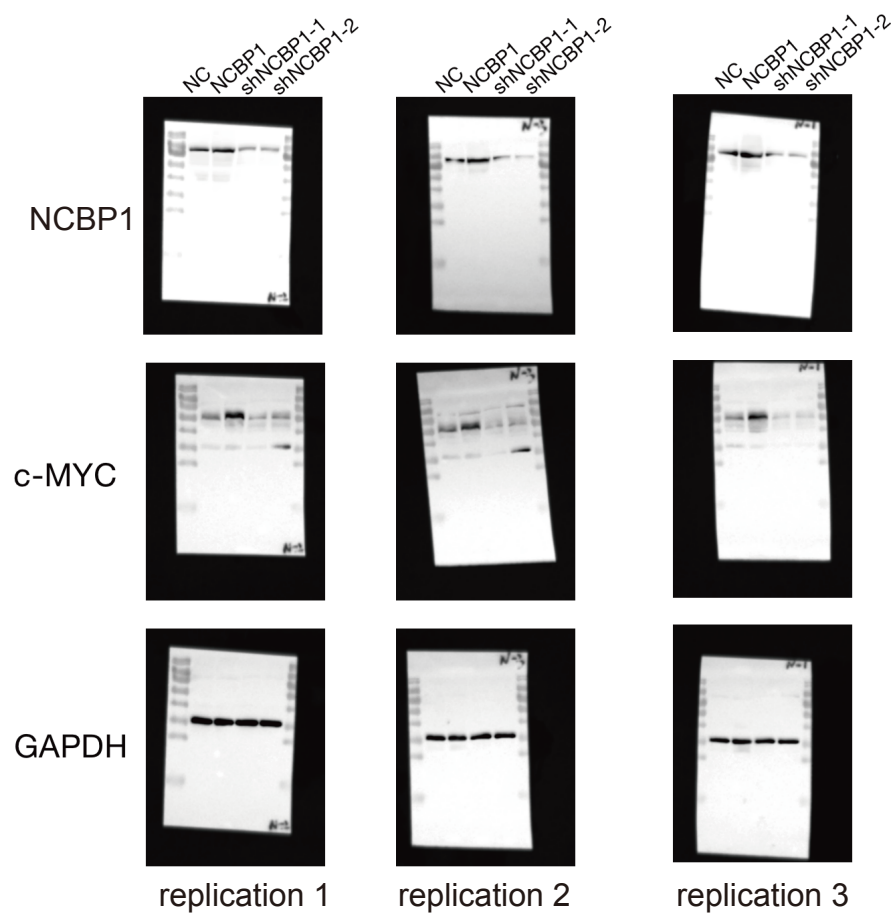

**Supplementary Figure 6. Full-length gels and blots.**  
A-B, Full-length gels of figure S2H,J.

Supplement: Supplementary file 6 — Supplementary Information 6. [file 41598_2023_35777_MOESM6_ESM.pdf]
