# Supplementary material for: NCBP1 enhanced proliferation of DLBCL cells via METTL3-mediated m6A modification of c-Myc
Source: Sci Rep. 2023 May 27;13:8606. doi: 10.1038/s41598-023-35777-2 (PMC10224985; doi:10.1038/s41598-023-35777-2)
Supplement: Supplementary file 5 — Supplementary Information 5. [file 41598_2023_35777_MOESM5_ESM.pdf]

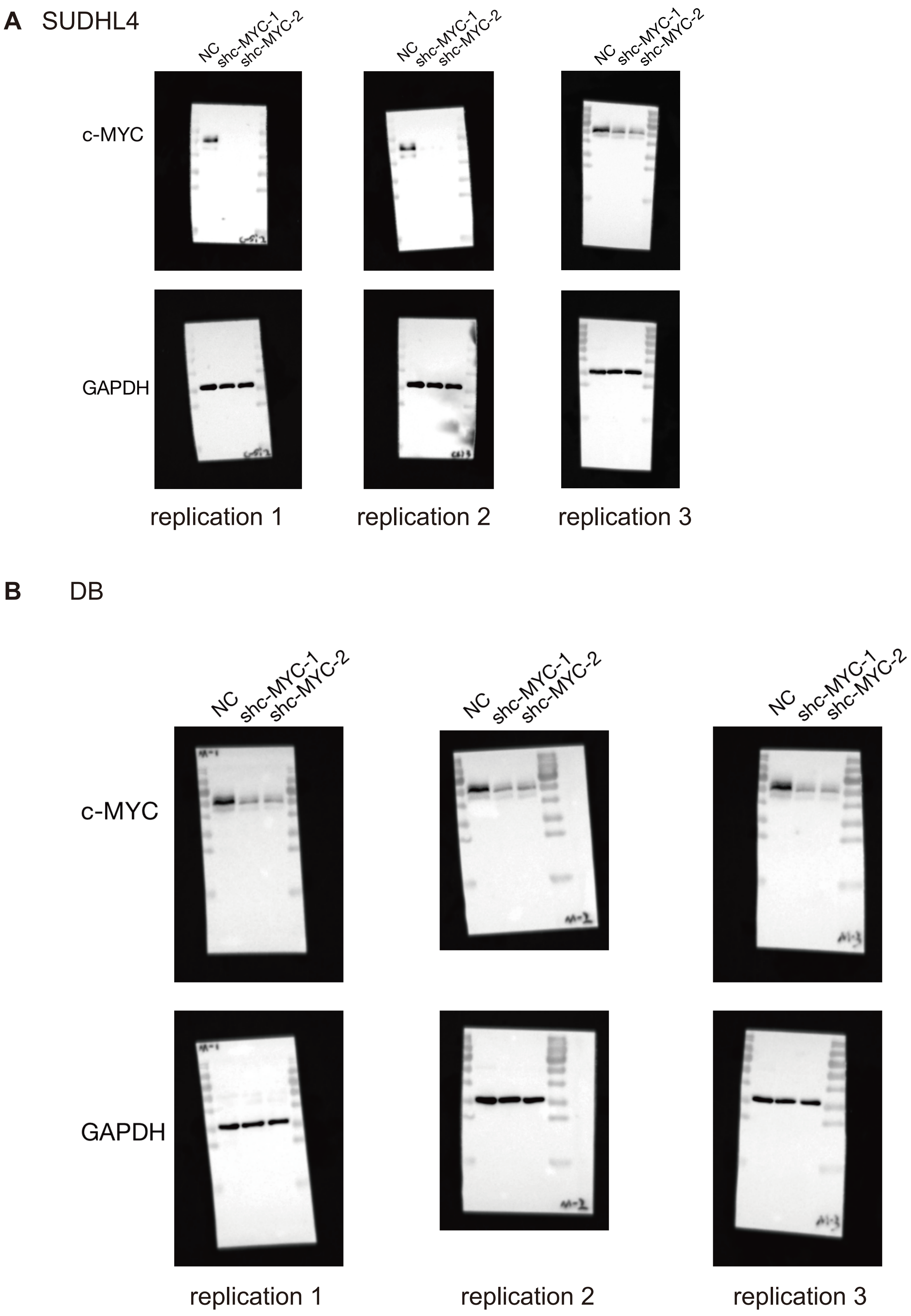

Supplementary Figure 5. Full-length gels and blots.
A-B, Full-length gels of figure 5E and S2D.
