# Supplementary material for: NCBP1 enhanced proliferation of DLBCL cells via METTL3-mediated m6A modification of c-Myc
Source: Sci Rep. 2023 May 27;13:8606. doi: 10.1038/s41598-023-35777-2 (PMC10224985; doi:10.1038/s41598-023-35777-2)
Supplement: Supplementary file 7 — Supplementary Information 7. [file 41598_2023_35777_MOESM7_ESM.docx]

Table 1 PCR primers

NCBP1 forward primer:5’-GGC TGC AGC AGA TCT TCC TA-3’,

NCBP1 reverse primer:5’-TCT CCA GGG TCA CCA TGT ACT-3’,

METTL3 forward primer:5’-CGT ACT ACA GGA TGA TGG CTT TC-3’,

METTL3 reverse primer:5’-TTT CAT CTA CCC GTT CAT ACC C-3’,

MYC forward primer: 5’-CCT CCA CTC GGA AGG ACT ATC-3’,

MYC reverse primer: 5’-TGT TCG CCT CTT GAC ATT CTC-3’,

GAPDH forward primer：5’-TCA TGG GTG TGA ACC ATG AGA A-3’,

GAPDH reverse primer: 5’-GGC ATG GAC TGT GGT CAT GAG-3.’
