# Supplementary material for: NCBP1 enhanced proliferation of DLBCL cells via METTL3-mediated m6A modification of c-Myc
Source: Sci Rep. 2023 May 27;13:8606. doi: 10.1038/s41598-023-35777-2 (PMC10224985; doi:10.1038/s41598-023-35777-2)
Supplement: Supplementary file 8 — Supplementary Information 8. [file 41598_2023_35777_MOESM8_ESM.docx]

Table 2 Lentiviral sequence

NCBP1(HOMO)siRNA CCUGAGAAGCUGACAAUUUTT AAAUUGUCAGCUUCUCAGGTT

NCBP1(HOMO)siRNA CCAUGAUUCGUCAACUUAATT UUAAGUUGACGAAUCAUGGTT

METTL3siRNA CUCAGUGGAUCUGUUGUGAUA UAUCACAACAGAUCCACUGAG

METTL3siRNA GCUGCACUUCAGACGAAUUAU AUAAUUCGUCUGAAGUGCAGC

MYC(HOMO)siRNA GCUUGUACCUGCAGGAUCUTT AGAUCCUGCAGGUACAAGCTT

MYC(HOMO)siRNA GGAAGAAAUCGAUGUUGUUTT AACAACAUCGAUUUCUUCCTT
